# The Military Status of the Medical Profession

**Published:** 1918-04

**Authors:** 


**A Monthly Review of Medicine and Surgery**

EDITOR AND PUBLISHER, DR. A. L. BENEDICT, 228 Summer St., corner of Elmwood Ave., Buffalo, (Address for all communications. Please make personal and telephone calls before 1 P. M.)

Third, in age, on the western continent. Independent, but supports professional organizations. News limited mainly to Western N. Y. and Penn.

Subscription $2.00 a year; $3.00 for two years in advance. Changes and errors in address or failure or duplication of delivery, should be reported immediately.

Yearly Volume 73. APRIL, 1918. Number 9

### The Military Status of the Medical Profession.

It may be claimed that our profession is unique among occupations, in regard to the honor shown it and the demands made upon it by the military authorities of the nation. While, both for strictly military appointment and for various services needed by the government, the special training and ability of individuals is duly considered, there is, in general, no official recognition of vocation, license to engage in a certain line of work or technical graduation, except for medicine, including dentistry, veterinary medicine and nursing, which are made auxiliary branches of the medical service under the control of the Surgeon General. Chaplains are, to a large degree, analogous, but there are considerable differences as to status in the military organization, as to the inforcement of demands upon the clergy for military service, in regard to the insistence upon technicalities of institutional training, etc. A very practical difference exists also in the fact that while there are even more clergymen of all kinds than physicians, and especially in proportion to the number of the population requiring their services, probably not more than a tenth as many will be required for the army and navy. Thus urgent problems of demand and supply will hardly be possible. Again, the peculiarly personal relation between clergy and laymen is such that problems as to maintenance after the war will not be so pressing as for the medical profession, and this fact has an important bearing on the problem of demand and supply.

It might be argued at first thought that an analogous condition exists with regard to the Judge Advocate General's Dept., but, while the emergent appointments to this Dept. will undoubtedly be drawn from the legal profession, we do not understand that the existing regular army officers are necessarily lawyers in the professional sense, their number (13 last year, with no enlisted personnel) is and will be small and, up to recently, the requirements for admission to the law have been very informal in comparison with those which have applied to the medical profession for so many years as to include almost every extant member of that profession. Somewhat the same reply might be made to a similar objection on behalf of the Corps of Engineers. For the other special departments and corps of the Army and Navy, as well as for the line, no claim of technical institutional training, possession of a degree and of a license, can be raised except as a quibble of words, to establish an analogy to the status of the medical profession.

The unique position of our profession is one to be proud of. It also bears heavy responsibilities. As has already been pointed out, the military force at present contemplated or, at least, publicly announced, demands about 15% of the entire medical profession, without regard to sex, age, physical condition or adequate reasons for exemption. It demands at least 25% of the actual practicing profession theoretically available. If the normal militia strength of the nation should be called upon (approximately 10%, 10 millions) probably every practically available physician would be called into service. This statement does not include the men already enrolled in the Army and Navy and the corresponding organized militias, on a peace footing, nor the physicians already employed by the government in auxiliary capacities. It is said to be literally true for France, and is probably either literally or practically true for the other continental belligerents, that every physician is now a military surgeon, though, of course, many are detailed for absolutely necessary service to the civil population.

Any such statements imply that the demands upon our profession, for all the countries involved, are and will continue to be nearly or quite ten times as great as upon the population generally, unless, of course, the maximum man power is called into service. They also imply that, even at the outset, the age limits and exemptions for the population generally cannot apply to the medical profession. Under present conditions, the average age at which a man enters the medical profession, is 26-27 if interne service is included. The number of physicians under 25 is very small while, absolutely or relatively, the number corresponding to the general draft age limits is scarcely half that contained in any similar average group of the male population.

This demand on the profession has been very nearly met by the volunteer system, and we trust that it will be fulfilled on this basis. But let us not be blind to the fact that, behind the volunteer system is the potential necessity and, therefore the potential power of the draft. Such a draft, if the full military strength of the nation should unfortunately be required, could scarcely be termed selective. In such an event it will be sweeping.

Right here, an extremely interesting problem arises. If urgent need arises, what will be the status of the non-practicing physician? We do not mean the man retired on account of full age, but those voluntarily retired at earlier ages, those who are teaching, conducting sanitariums, manufacturing drugs or acting as agents, or engaged in such extra-medical vocations as teaching, real estate, law, selling automobiles, making candy, running theatres, holding political offices, dealing in tea and spices, etc? How will this status differ as to whether the man was merely graduated, whether he was graduated at a time when graduation with or without formal registration constituted a license to practice, whether he was also formally licensed by some state board, whether he never practiced at all, or only as an interne or as an assistant to another physician, or in some official capacity, whether his status was ethical or unethical, whether his abandonment of practice was recent or remote? It is easier to raise such questions than to answer them, but they are no more impracticable than the establishment of principles relative to aerial navigation a few years ago, than the establishment of the Medical Reserve or any other question of preparedness against an unrealized emergency.

